# Symptoms of Poststroke Depression among Stroke Survivors: An Appraisal of Psychiatry Needs and Care during Physiotherapy Rehabilitation

**DOI:** 10.1155/2016/5646052

**Published:** 2016-04-13

**Authors:** Sam Chidi Ibeneme, Canice Chukwudi Anyachukwu, Akachukwu Nwosu, Georgian Chiaka Ibeneme, Muideen Bakare, Gerhard Fortwengel

**Affiliations:** ^1^Department of Medical Rehabilitation, Faculty of Health Sciences & Technology, College of Medicine, University of Nigeria, Enugu Campus, Enugu 40024, Nigeria; ^2^German UNESCO Unit on Bioethics, Fakultat III-Medien, Information und Design, Hochschule Hannover-University of Applied Sciences and Arts, 30539 Hannover, Germany; ^3^National Institute for Sports, Surulere, Lagos 101283, Nigeria; ^4^Department of Nursing Sciences, Faculty of Health Sciences & Technology, College of Medicine, Ebonyi State University, Abakaliki 5480214, Nigeria; ^5^Federal Neuro-Psychiatric Hospital, New Haven, Enugu 400221, Nigeria

## Abstract

*Purpose*. To identify stroke survivors with symptoms of poststroke depression and the extent of psychiatry needs and care they have received while on physiotherapy rehabilitation.* Participants*. Fifty stroke survivors (22 females and 28 males) at the outpatient unit of Physiotherapy Department, University of Nigeria Teaching Hospital, Enugu, who gave their informed consent, were randomly selected. Their age range and mean age were 26–66 years and 54.76 ± 8.79 years, respectively.* Method*. A multiple case study of 50 stroke survivors for symptoms of poststroke depression was done with Beck's Depression Inventory, mini mental status examination tool, and Modified Motor Assessment Scale. The tests were performed independently by the participants except otherwise stated and scored on a scale of 0–6. Data were analyzed using *Z*-test for proportional significance and chi-square test for determining relationship between variables, at *p* < 0.05.* Results*. Twenty-one (42.0%) stroke survivors had symptoms of PSD, which was significantly dependent on duration of stroke (*χ* ^2^ = 21.680, df = 6, and *p* = 0.001), yet none of the participants had a psychiatry review.* Conclusions*. Symptoms of PSD may be common in cold compared to new cases of stroke and may need psychiatry care while on physiotherapy rehabilitation.

## 1. Introduction

The changing patterns of dietary intake, lifestyle, and living conditions in addition to the global increase in the ageing population have been associated with a rise in noncommunicable diseases such as stroke [1]. Stroke has been on the rise in sub-Saharan Africa within the last three decades and has attracted a lot of scientific attention in recent times [2]. Stroke is a “condition with rapidly developing clinical signs of a focal disturbance of cerebral function, of more than 24-hours duration of symptoms or leading to death, with no apparent cause other than that of vascular origin” [3]. “Included within this definition are most cases of cerebral infarction, cerebral haemorrhage and subarachnoid haemorrhage, but deliberately excluded are those cases in which recovery occurs within 24 hours.” “These later cases are designated ‘transient ischemic attacks' (TIA), because they are often a harbinger of completed stroke, and have received considerable attention over the past two decades” [4]. This definition has been updated by the Stroke Council of the American Heart Association/American Stroke Association such that “central nervous system infarction is defined as brain, spinal cord, or retinal cell death attributable to ischemia, based on neuropathological, neuroimaging, and/or clinical evidence of permanent injury. Ischemic stroke specifically refers to central nervous system infarction accompanied by overt symptoms, while silent infarction by definition causes no known symptoms. Stroke also broadly includes intracerebral hemorrhage and subarachnoid hemorrhage” [5].

Generally, stroke should be considered as a negative life event, which may lead to depression. Depression in many ways resembles the grief/mourning that follows bereavement and often evokes feelings of low self-esteem, guilt, self-reproach, withdrawal from interpersonal contact, and somatic symptoms such as eating and sleeping disturbances [6]. Usually, onset of depression in patients might be determined by the interaction between personality factors and the severity of the negative physical, psychological, and social consequences of stroke [7].

Recently, depression is recognised as a common complication of stroke that may result in high morbidity and mortality rates [2]. Depression can impede recovery and has been linked with poorer treatment outcomes and increased length of stay in hospital [7]. Since depression can reduce motivation and worsen treatment outcome, stroke survivors on rehabilitation should be routinely screened in order to identify individuals at risk and target appropriate intervention for effective rehabilitation outcome. Therefore, there is a need to appraise the psychiatry needs of stroke survivors and the sufficiency of the available clinical practice in meeting these needs for effective rehabilitation outcome. Thus, this study is aimed at identifying stroke survivors that are more likely to have symptoms of poststroke depression and the extent of psychiatric care they have received while on physiotherapy rehabilitation. It is predicted that there is an association between symptoms of depression and age, marital status, occupation, gender, location of hemispheric lesion, and complications, respectively.

## 2. Materials and Methods

### 2.1. Research Design

A multiple case study of stroke survivors on physiotherapy rehabilitation was carried out to determine their psychiatry needs and relevant services targeted towards addressing those needs for improved treatment outcome.

### 2.2. Area of Study

*Setting*. This study was carried out at the outpatient clinic (Adult Neurology Unit) of the Physiotherapy Department, University of Nigeria Teaching Hospital (UNTH), Ituku-Ozalla.

### 2.3. Participants Selection

Fifty stroke survivors (22 females and 28 males), who gave their informed consent, were randomly selected from those that had current physiotherapy appointments using balloting method.

### 2.4. Selection Criteria

#### 2.4.1. Inclusion Criteria

Inclusion criteria are as follows:

- Only participants that satisfied WHO's [2] definition of stroke must be selected.
- Only stroke survivors with intact cognition (as determined using mini mental status examination) must be involved.
- Only stroke survivors between 26 and 66 years of age must be involved.
- Only stroke survivors who receive physiotherapy treatment at the University of Nigeria Teaching Hospital, Ituku-Ozalla, must be involved.

#### 2.4.2. Exclusion Criteria

Exclusion criteria are as follows:

- All those unable to communicate meaningfully (because of severe aphasia or cognitive dysfunction) must be excluded.
- All those with TIA (transient ischemic accident) must be excluded.
- All those with other pathologies of neurological or orthopaedic nature must be excluded.

### 2.5. Participants Description

The participants were comprised of male and female stroke survivors that are currently on physiotherapy rehabilitation at the Physiotherapy Department of the University of Nigeria Teaching Hospital, Ituku-Ozalla, at the time of this study.

### 2.6. Ethical Approval: Ethical Consideration

Ethical approval from the Health Research Ethics Committee of the University of Nigeria Teaching Hospital, Ituku-Ozalla, was obtained alongside a supervision approval from a Consultant Psychiatrist. All the participants gave their informed consent to participate in the study after the purpose was explained to them. They were also assured of the confidentiality of the information they provide. It was made clear to the participants that they have the right to refuse to participate or to withdraw at any stage of the project, and these rights were respected all through the research procedure.

### 2.7. Research Instrument

#### 2.7.1. Modified Motor Assessment Scale (MMAS)

The Modified Motor Assessment Scale (MMAS) was used to assess motor recovery in stroke survivors with an intrarater reliability of 0.83 to 1.00 with median of 0.97, using Spearman's rank-order correlation coefficient.

### 2.8. Beck's Depression Inventory

Beck's Depression Inventory (BDI-II) is a psychometric test, which was used for measuring the severity of depression [8], and has been validated among Nigerians [9] with an “internal consistency of 0.86, reliability of 0.93 [10], sensitivity of 0.91, specificity of 0.97, positive predictive value (PPV) of 0.88, and negative predictive value (NPV) of 0.98” [9]. Its cutoff score is 18 and above, with high rating for content validity [10].

Other instruments used for the study are comb, spoon, tea cups, and jelly box or precision box as well as stop watch, which was used for timing Motor Assessment Scale measurement.

### 2.9. Data Analysis

The data collected for this study were analyzed using Statistical Package for Social Science (SPSS) 16.0, Windows version. The results were presented in Tables 1, 2, and 3. Inferential statistics was utilized, including *Z*-test, to determine proportional significance, and chi-square test as a measure of association between the variables. Alpha was set at 0.05.

## 3. Results and Discussion

### 3.1. Demographic Variables and Symptoms of Poststroke Depression

Results are presented in Tables 1–3 and include the symptoms of depression in stroke survivors (Table 1) in relation to some sociodemographic (Table 2) and clinical variables (Table 3). It was observed that, out of the 50 stroke survivors, 21 (42.0%) had symptoms of various levels of depression, whereas 29 (58.0%) had no clinical indication of depression (Table 1). In fact, not only were symptoms of depression recorded in almost half of the participants, but it was further observed (Table 1) that, out of the 21 stroke survivors that had symptoms of depression, 10 (47.62%) had symptoms of moderate depression, while 11 (52.38%) had symptoms of borderline clinical depression.

Overall, the proportion of those that had depression was not significant. Also, statistical analysis of the data showed that symptoms of poststroke depression are not dependent on sociodemographic variables (gender, marital status, occupation, and age). From these results it appears that almost half (42%) of the stroke survivors had symptoms of PSD. These findings agree with previous observations that depression is a common complication of stroke, leading to increased morbidity and mortality [6], and occurs in approximately 40–50% of patients [11]. In fact, it has been observed that stroke survivors may experience a number of psychological sequalae including depression [11]. Overall, the trends from the results of our study suggest that there is a likely relative but not significant influence of age, duration of stroke, living with spouse, type of employment, gender, hemispheric asymmetry, and complications on mood of stroke survivors. Thus, there was no significant association between these variables and symptoms of PSD, respectively.

The results further show that 10 (45.46%) out of 22 females and 10 (35.71%) out 28 males had symptoms of depression (Table 2). Also, 18 (90%) out of 20 married stroke survivors had symptoms of depression, whereas 3 (25%) out of 12 unmarried stroke survivors had symptoms of depression. It was observed that 11 (28.95%) of the 38 stroke survivors that were not self-employed (civil servants and banker) had depression, whereas 9 (40.91%) of the 22 self-employed (drivers, artisans, farmers, housewives, retirees, and petty trader) stroke survivors had depression. Furthermore, 14 (60.87%) of the 23 young/middle-aged stroke survivors (aged 27–56 years) had symptoms of depression, whereas 8 (29.63%) of the 27 geriatric stroke survivors had symptoms of depression. Within the young/middle-aged stroke survivors, it was further shown that all (i.e., 2 or 100%) of those within 27–36 years had symptoms of depression, whereas 3 (50%) of the 6 stroke survivors within 37–46 years had symptoms of depression. Also, 9 (60%) of the 15 stroke survivors that were aged between 47 and 56 years had symptoms of depression. However, only 8 (42.11%) out of 27 older adult (aged 57–66 years) stroke survivors had symptoms of depression. Invariably, the distribution of the symptoms of poststroke depression age group is 9.09% (27–36 years), 3.64% (37–46 years), 40.91% (47–56 years), and 36.36% (57–66 years). In addition, it was shown that 11 (40.74%) of the 27 patients that presented with left-sided hemiplegia (i.e., lesion of the right cerebral hemisphere) had symptoms of poststroke depression, whereas only 9 (39.13%) of the 23 stroke survivors that presented with right-sided hemiplegia (i.e., lesion of the left cerebral hemisphere) had symptoms of depression. These findings may imply that middle-aged adults (47–56 years) and older middle-aged adults (57–66 years) were more likely to have symptoms of depression than others and may relate to how the various age groups respond to the psychological impact of associated functional impairment/loss of independence in activities of daily living associated with stroke. Such functional limitation may affect perception of self, especially self-image and self-worth, eventually leading to depression [6]. This finding agrees with previous observations from studies done outside sub-Saharan Africa, which indicate that PSD is associated with old age [12, 13] rather than young age. In fact, substantial evidence from such studies has shown that depression is a common condition in the elderly and that the incidence of stroke increases with age. Nevertheless, Sharpes et al. [14] observed that, irrespective of age, anyone can react to adversity with depressive mood and may explain why some reasonable proportion of both young and old stroke survivors presented with symptoms of depression in this study. Apparently, the results suggest that symptoms of depression are not dependent on age and gender differences.

The fact that almost all (90%) of stroke survivors living with their spouses (married) seem to have symptoms of depression unlike those that do not live with their spouses may relate to a state of frustration due to the inability of a stroke survivor to support his/her spouse financially, emotionally, physically, socially, and with household chores, due to incapacitation by stroke. The net psychological effects may eventually trigger depression. Thus, psychiatric review may target threats to effective family functioning, because it may be fractured under the stress of financial burden, physical burn-out, and emotional drain of spouses who act as caregivers. However, it appears from this study that a family that functions at the required level of socialization (trust, good communication skills, care, tolerance, etc.) is likely to deal with the needs of stroke survivors better than others and thus buffer the predisposing events likely to trigger symptoms of PSD.

The findings of this study further indicated that, among 40 stroke survivors with health complications (including 39 hypertensive and 1 dementia cases), 18 (46.15%) of the 39 stroke survivors with hypertension had symptoms of depression, whereas the only stroke survivor with dementia had no such symptoms. In essence, symptoms of depression were found in 45% of those with complications (i.e., those with hypertension and dementia, *n* = 40) and in 2 (20%) of the 10 stroke survivors who did not have any complications, but none of them had a psychiatry review in the course of physiotherapy rehabilitation. Statistical analysis of the results shows that depression in stroke patients is independent of complications (hypertension and dementia). The results show that about 57.14%, 77.78%, and 100% of the stroke survivors with 1–3-year, 4–6-year, and 7–9-year duration of occurrence had symptoms of poststroke depression, respectively. Statistical analysis shows that depression is dependent on duration of stroke (*χ* ^2^ = 21.680, df = 6, and *p* = 0.001). The implications of these findings are that most cases of stroke survivors with lesions on the right compared to left cerebral hemisphere (which are presented as left-sided hemiplegia) are more likely to have symptoms of PSD. This observation agrees with a previous report, which suggests that incidence of PSD was higher among stroke survivors with left-sided hemiplegia compared to that among stroke survivors with right-sided hemiplegia [13]. In contrast, another study revealed that depression was significantly correlated only with the side of intracerebral lesion, with patients with the right-hemiplegia being more depressed [15]. However, previous authors have made interesting observations that seem to suggest that the association between PSD and intracerebral lesion location depends on the duration of stroke in the population studied by an investigator. For instance, it was indicated that many factors influence the interval from a stroke to the appearance of depression [16, 17]. Thus, during the weeks immediately after a stroke, the intracerebral lesion's location seems to be the most important factor in determining PSD, but 6–12 months after a stroke, other factors such as quality of social integration and severity of functional/cognitive impairment seem to be more important in determining mood disorders [15]. Therefore, any association between location of intracerebral lesion and PSD must be interpreted in the context of the duration of the stroke.

Interestingly, this study revealed that majority of the stroke survivors that were yet to recover after 3 years of onset had symptoms of depression unlike the observation in those with less than 3 years of onset. The results agree with previous findings [15] contrary to our earlier prediction. Invariably, it seems that the quicker the social integration and functional and cognitive recovery of stroke survivors, the lesser the possibility of having symptoms of PSD, and* vice versa*. This may explain why symptoms of PSD were recorded among a high proportion of those that did not recover from stroke after >3 years of onset compared to <3 years of onset. Another study characterized “the natural course of major depression after stroke” and observed that “there is spontaneous remission typically 1 to 2 years after stroke” [10]. However, it was also noted that, in a few cases, depression becomes chronic and may persist more than 3 years following stroke. In our study, it was observed that symptoms of poststroke depression were significantly dependent on the duration of stroke.

These findings underlie the need for quick psychiatric and physiotherapy intervention for effective rehabilitation of stroke survivors in order to take advantage of the period of neural plasticity and effect faster recovery of function. This is reasonable since prevalence of depression after 3 years of onset of stroke can impede the process of rehabilitation and has been associated with poorer outcomes and increased length of stay in hospital [6]. In fact, it has been observed that the pathogenesis of depression derives from disturbance of neuroplasticity and reduced neurogenesis [18]; therefore timely prescription of antidepressants by psychiatrists in stroke management may hold significant promise in relieving the possibility of PSD through its neuroprotective effects on brain plasticity and neurogenesis [19, 20]. Surprisingly, this practice is not currently applicable at the tertiary hospital where this study was conducted and, perhaps, likewise, other hospitals. Thus, none of the stroke survivors that were studied had psychiatry assessment for symptoms of PSD. Therefore, it is important that psychiatry review and preventive use of antidepressants be prescribed, where necessary, at onset of stroke, [18] prior to physiotherapy management or alongside it, for a better treatment outcome.

## 4. Conclusion

Generally, the pattern of results presented in the contingency Table 1 appears compatible but does not significantly agree with the predictions made for this study. The findings of this study point to the likelihood that stroke has a broad negative psychological impact on the personality of a reasonable spectrum of survivors. Surprisingly, the clinicians have paid little attention to this fact, such that none of the stroke survivors in this study was either assessed by a psychiatrist or referred for psychiatry review since poststroke depression, which may arise, is known to impede rehabilitation.

## Figures and Tables

**Table 1:** Distribution of depression among stroke patients (*N* = 50).

BDI scale	Mood	Number of patients	Percentage (%)	*Z*-value	Hospital record of assessment/treatment of PSD
1–10	Normal	15	30.0	−2.828	Nil
11–16	Mild mood disturbance	14	28.0	−5.259	Nil
17–20	Borderline clinical depression	11	22.0	−3.959	Nil
21–30	Moderate depression	10	20.0	−4.243	Nil
>30	Severe depression	0	0.0	—	Nil

Total		**50**	**100**		

BDI = Beck's Depression Inventory; PSD = Poststroke depression; *Z*_tab_ = 1.96 at *p* < 0.05.

**Table 2:** Relationship between depression and sociodemographic variables (age, gender, marital status, and occupation; *N* = 50).

Sociodemographic characteristics	Nondepressed	Depressed	*χ* ^2^	*p*
Age				
27–36 years	0	2	12.560	0.945
37–46 years	3	3		
47–56 years	6	9		
57–66 years	19	8		
Gender				
Female	12	10	0.486	0.486
Male	18	10		
Marital status				
Divorced	1	0	3.932	0.415
Married	20	18		
Separated	3	1		
Single	3	1		
Widowed	2	1		
Occupation				
Artisan	0	1	7.332	0.395
Banker	1	3		
Civil servant	16	8		
Driver	1	0		
Farmer	4	2		
Housewife	1	2		
Petty trader	1	0		
Retiree	6	4		

*χ* ^2^ = chi-square.

**Table 3:** Relationship between depression and clinical demographic variables (complications and presenting pattern; *N* = 50).

	Variables studied	Nondepressed	Depression	*χ* ^2^	*p* value
Location of cerebral lesion	Left	16	11	0.013	0.909
	Right	14	9		

Complications	Dementia	1	0	3.459	0.177
	Hypertension	21	18		
	Nil	8	2		

Duration of occurrence	1–3	21	12	21.680	0.001^*∗*^
	4–6	9	7		
	7–9	0	1		

*χ* ^2^ = chi-square; *∗* indicates significance at *p* < 0.05.
